# Supplementary material for: Serotype specific epitopes identified by neutralizing antibodies underpin immunogenic differences in Enterovirus B
Source: Nat Commun. 2020 Sep 4;11:4419. doi: 10.1038/s41467-020-18250-w (PMC7474084; doi:10.1038/s41467-020-18250-w)
Supplement: Supplementary file 1 — Supplementary Information [file 41467_2020_18250_MOESM1_ESM.pdf]

Supplementary Information

Serotype specific epitopes identified by neutralizing antibodies underpin immunogenic differences in Enterovirus B

Wang et al.

This file contains Supplementary Figures 1-4 and Supplementary Tables 1-3.

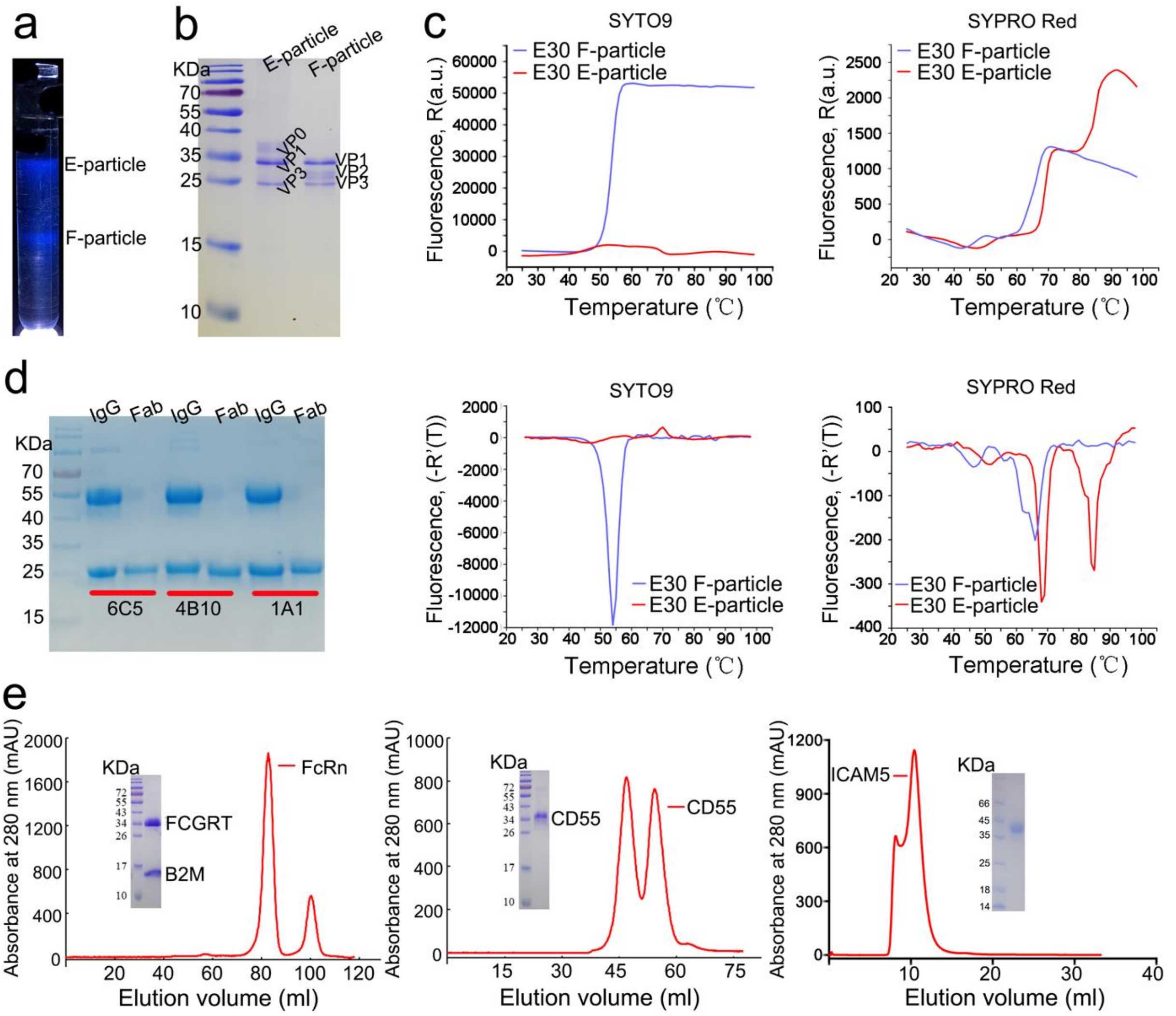

Supplementary Figure 1

Purification of E30, 6C5 Fab and 4B10 Fab. (a) Sucrose density gradient ultracentrifugation (from 15% to 45%) for the purification of E30, as described in Methods. Two marked bands, i.e., the top with an absorbance ratio of 1.7 mainly containing the F-particles, and the bottom with an absorbance ratio of 0.7, containing the E-particles could be identified. **(b)** SDS-PAGE analysis for viral protein composition. The estimated molecular weights of VP0, VP1, VP2, VP3 of E30 are 36 kDa, 32 kDa, 29 kDa, 26 kDa, respectively. **(c)** Stabilities of the purified intact E30 F-particle and E-particle at pH = 7.4 were analyzed by thermofluor assay. We used SYTO9 (left) and SYPRO Red (right) to detect the exposed viral RNA and hydrophobic residues, respectively. **(d)** SDS-PAGE analysis of 6C5 IgG, 4B10 IgG and 1A1 IgG, as well as their corresponding Fab fragments cleaved from the whole antibodies using Pierce FAB preparation kit (Thermo Scientific). **(e)** The SDS-PAGE and gel filtration of the receptors - FcRn (left), CD55 (middle) and ICAM5 (right).

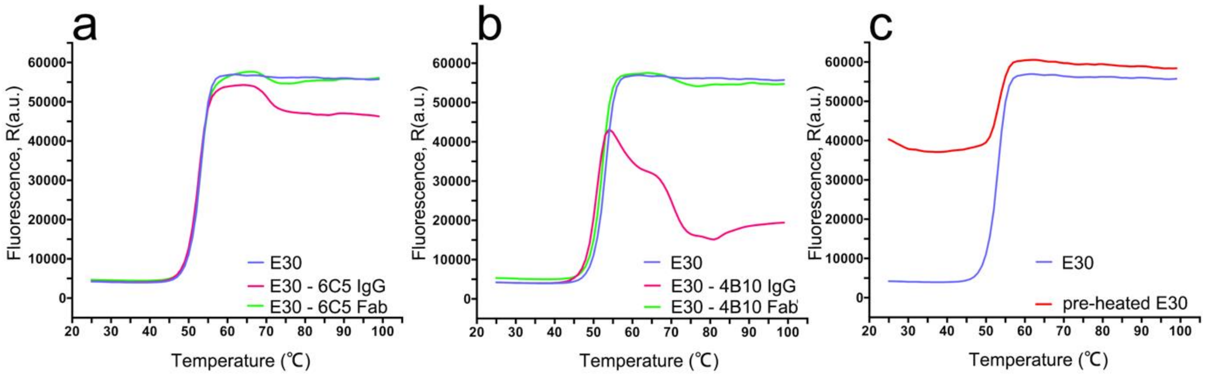

Supplementary Figure 2

RNA-releasing Analysis.

To characterize the stability of E30 upon the binding of 6C5 IgG/Fab **(a)** or 4B10 IgG/Fab **(b)**, particle stability thermal release assays were performed with the dye SYTO9 to detect RNA exposure. **(c)** E30 and heat-treated E30 (at 55 ℃ for 5 min prior to RNA detection) act as negative and positive controls, respectively. The raw fluorescence traces are shown. The higher SYTO9 fluorescence signal at the room temperature for the heat-treated E30 implies that RNA genome of heat-treated particles is accessible to the dye at room temperature, whereas RNA in E30 or E30-6C5 or E30-4B10 mixtures only becomes accessible at 51-55 ℃.

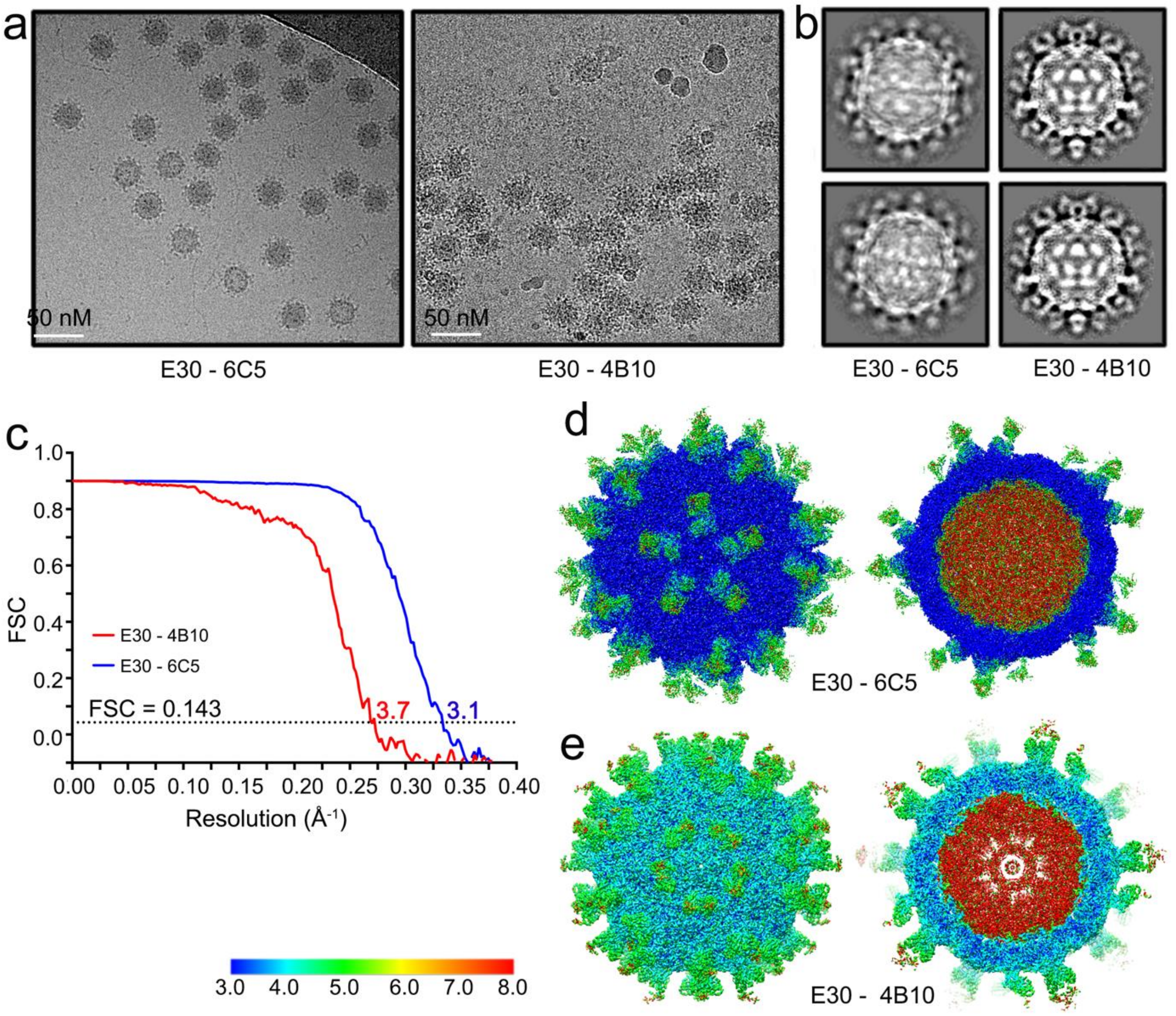

Supplementary Figure 3

Cryo-EM Images and Map Resolution Evaluation. (a) Cryo-EM micrographs of E30-6C5-complex (left) and E30-4B10-complex (right). **(b)** Representative classes from 2D classification of these two groups of complexes in Relion 3.0[1]. **(c)** Gold-standard Fourier shell correlation (FSC) curves of the final maps of the complexes[2]. Map resolution assessment of the complexes – E30-6C5 **(d)** and E30-4B10 **(e)** with color indicated below.

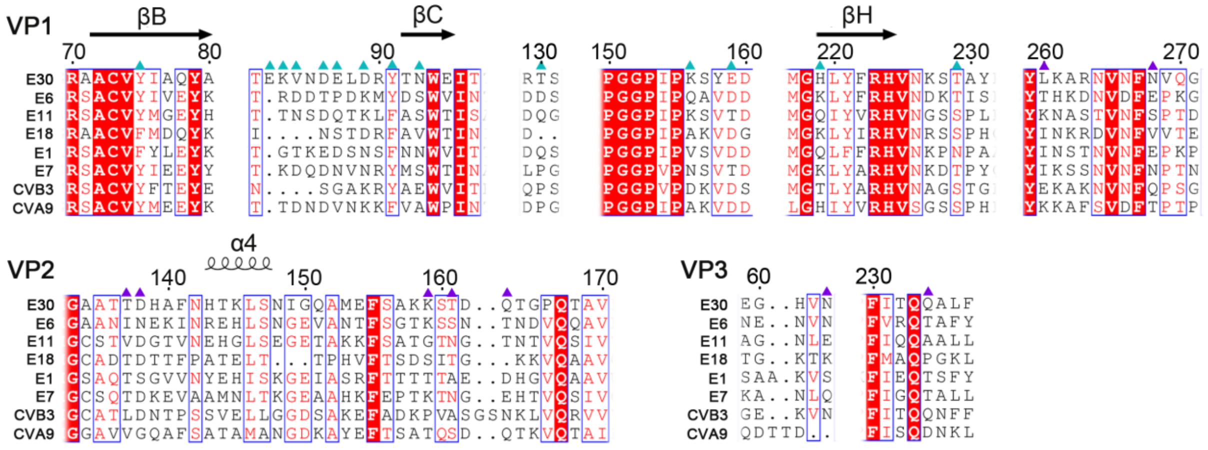

Supplementary Figure 4

Sequence Alignment of Fab-binding regions. Espript[3] representation of sequence alignment of regions in the vicinity of the epitopes targeted by 6C5 and 4B10. Residues directly interact with 6C5 or 4B10 are marked with cyan triangles or purple triangles, respectively.

Supplementary Table 1 **Statistics for Cryo-EM imaging, data processing and refinement of models.**

Name	F-particle in complex with 6C5	E-particle in complex with 6C5	F-particle in complex with 4B10
**Data collection**			
Microscope		FEI Titan Krios	
Camera		Gatan K2	
Voltage (kV)		300	
Total dose ($e^-/Å^2$)		30	
Symmetry imposed		I	
Micrographs (total)	1,661		998
Micrographs (used)	1,454		719
Particles selected	16,488		18,328
Particles included in final reconstruction	10,127	2,120	1,841
**Reconstruction**			
Sampling, Å per pixel	1.320	1.320	1.313
Defocus range (μm)	1.2 - 2.5	1.2 - 2.5	1.2 - 2.5
Resolution (Å) (FSC = 0.143 criterion)	3.1	3.4	3.7
**Model Refinement**			
Clash score	5.00	4.00	5.00
Rotamer outliers (%)	0.98	0.31	0.47
Molprobity score	1.59	1.80	1.79
**Ramachandran statistics (%)**			
Most favored (%)	94.88	90.51	91.38
Allowed (%)	5.12	9.40	8.54
Outliers (%)	0	0.09	0.08
**R.m.s.deviations**			
Bond lengths (Å)	0.006	0.007	0.005
Bond angles (°)	0.900	0.998	0.921

Supplementary Table 2 **List of interactions between E30 and 6C5.**

E30			6C5			
Location	Domain	Residues	Residues	Domain	Distance	Type of interaction
VP1	BC loop	E82 [OE1]	S55 [OG]	HCDR2	3.87	Hydrogen bond
		K83 [O]	W33 [NE]	HCDR1	2.73	
		K83 [NZ]	L100 [O]	HCDR3	3.29	
		K83 [NZ]	D99 [OD2]	HCDR3	3.21	
		D86 [O]	R101 [O]	HCDR3	2.88	
		D89 [OD2]	R101 [NH2]	HCDR3	2.99	
		Y91 [OH]	R101 [NE]	HCDR3	3.39	
		Y91 [OH]	R101 [NH2]	HCDR3	3.43	
	DE loop	T130 [OG1]	H54 [ND1]	HCDR2	3.23	
	EF loop	E159 [OE2]	Y32 [OH]	HCDR1	3.44	
	HI loop	T229 [O]	H54 [NE2]	HCDR2	2.94	
	BC loop	K83 [NZ]	D99 [OD2]	HCDR3	3.21	salt bridge
		D89 [OD2]	R101 [NE]	HCDR3	3.85	
		D89 [OD2]	R101 [NH2]	HCDR3	2.99	
	BC loop	V84 [O]	W90 [NE1]	LCDR3	3.46	hydrogen bond
		D86 [OD1]	H33 [NE2]	LFR2	3.92	
		E87 [OE1]	R30 [NH2]	LCDR1	3.79	
	B strand	Y75 [OH]	R30 [NH1]	LCDR1	3.66	
	C strand	N93 [OD1]	Y31 [OH]	LCDR1	3.14	
	EF loop	K156 [NZ]	Y4 8[OH]	LFR2	3.20	
	H strand	H219 [NE2]	Y51 [OH]	LCDR2	3.85	
	BC loop	D86 [OD1]	H33 [NE2]	LFR2	3.92	salt bridge
		E87 [OE1]	R30 [NH2]	LCDR1	3.79	

Supplementary Table 3 **List of interactions between E30 and 4B10.**

E30			4B10			
Location	Domain	Residues	Residues	Domain	Distance	Type of interaction
VP1	C-terminus	L260 [O]	Y96 [OH]	LCDR3	2.80	hydrogen bond
		N268 [OD1]	S52 [OG]	LCDR2	2.98	
		N268 [ND2]	S52 [OG]	LCDR2	3.47	
		N268 [OD1]	A97 [O]	LCDR3	2.68	
VP2	EF loop	T137 [OG1]	G98 [N]	LCDR3	3.65	
		D138 [OD1]	S99 [OG]	LCDR3	3.06	
		K159 [NZ]	A51 [O]	LCDR2	3.84	
		K159 [N]	K55 [NZ]	LCDR2	3.75	
		T161 [OG1]	K100 [NZ]	LCDR3	2.78	
VP3	N-terminus	N63 [OD1]	E54 [OE2]	LCDR2	2.43	
		N63 [ND2]	E54 [OE2]	LCDR2	1.74	
	C-terminus	Q234 [OE1]	Q27 [O]	LCDR1	3.92	
VP2	EF loop	Q163 [OE1]	S30 [OG]	HCRD1	2.33	

Supplementary References

1. Scheres, S. H. W. RELION: implementation of a Bayesian approach to cryo-EM structure determination. *J. Struct. Biol.* **180,** 519–530 (2012).
2. Scheres, S. H. W. & Chen, S. Prevention of overfitting in cryo-EM structure determination. *Nat. Methods* **9,** 853–854 (2012).
3. Robert, X. & Gouet, P. Deciphering key features in protein structures with the new ENDscript server. *Nucleic Acids Res.* **42,** W320–4 (2014).
