# Supplementary material for: Serotype specific epitopes identified by neutralizing antibodies underpin immunogenic differences in Enterovirus B
Source: Nat Commun. 2020 Sep 4;11:4419. doi: 10.1038/s41467-020-18250-w (PMC7474084; doi:10.1038/s41467-020-18250-w)
Supplement: Supplementary file 3 — Reporting Summary [file 41467_2020_18250_MOESM3_ESM.pdf]

nature research

Corresponding author(s): Xiangxi Wang

Last updated by author(s): Aug 5, 2020

Reporting Summary

Nature Research wishes to improve the reproducibility of the work that we publish. This form provides structure for consistency and transparency in reporting. For further information on Nature Research policies, see our Editorial Policies and the Editorial Policy Checklist.

Statistics

For all statistical analyses, confirm that the following items are present in the figure legend, table legend, main text, or Methods section.

n/a	Confirmed	
☐	☒	The exact sample size (n) for each experimental group/condition, given as a discrete number and unit of measurement
☐	☒	A statement on whether measurements were taken from distinct samples or whether the same sample was measured repeatedly
☒	☐	The statistical test(s) used AND whether they are one- or two-sided<br>*Only common tests should be described solely by name; describe more complex techniques in the Methods section.*
☒	☐	A description of all covariates tested
☒	☐	A description of any assumptions or corrections, such as tests of normality and adjustment for multiple comparisons
☒	☐	A full description of the statistical parameters including central tendency (e.g. means) or other basic estimates (e.g. regression coefficient) AND variation (e.g. standard deviation) or associated estimates of uncertainty (e.g. confidence intervals)
☒	☐	For null hypothesis testing, the test statistic (e.g. F, t, r) with confidence intervals, effect sizes, degrees of freedom and P value noted<br>*Give P values as exact values whenever suitable.*
☒	☐	For Bayesian analysis, information on the choice of priors and Markov chain Monte Carlo settings
☒	☐	For hierarchical and complex designs, identification of the appropriate level for tests and full reporting of outcomes
☒	☐	Estimates of effect sizes (e.g. Cohen's d, Pearson's r), indicating how they were calculated

Our web collection on statistics for biologists contains articles on many of the points above.

Software and code

Policy information about availability of computer code

Data collection	SerialEM
Data analysis	GraphPad Software, SEDFIT software, COOT, Phenix, MOTIONCORR, EMAN package, Gctf, Relion 1.4, CHIMERA, Pymol

For manuscripts utilizing custom algorithms or software that are central to the research but not yet described in published literature, software must be made available to editors and reviewers. We strongly encourage code deposition in a community repository (e.g. GitHub). See the Nature Research guidelines for submitting code & software for further information.

Data

Policy information about availability of data

All manuscripts must include a data availability statement. This statement should provide the following information, where applicable:

- Accession codes, unique identifiers, or web links for publicly available datasets
- A list of figures that have associated raw data
- A description of any restrictions on data availability

The atomic coordinates of E30 (F-particle)-6C5-complex, E30 (F-particle)-4B10-complex and E30 (E-particle)-6C5-complex have been submitted to the Protein Data Bank with accession numbers: 7C81, 7C80 and 7CMK, respectively. The cryo-EM density maps of E30 (F-particle)-6C5-complex and E30 (F-particle)-4B10-complex have been deposited in the Electron Microscopy Data Bank under accession codes: EMD-30304, EMD-30303 and EMD-30408, respectively. Source data are provided with this paper. Other data are available from the corresponding authors upon reasonable request.

Field-specific reporting

Please select the one below that is the best fit for your research. If you are not sure, read the appropriate sections before making your selection.

☒ Life sciences ☐ Behavioural & social sciences ☐ Ecological, evolutionary & environmental sciences

For a reference copy of the document with all sections, see nature.com/documents/nr-reporting-summary-flat.pdf

Life sciences study design

All studies must disclose on these points even when the disclosure is negative.

Sample size	Trial experiments done previously were used to determine sample size with adequate statistical power.
Data exclusions	No data were excluded from the analyses.
Replication	For each experiment, three attempts at replication were successful.
Randomization	Gender- and age-matched mice were randomly assigned to groups for in vivo experiments.
Blinding	The investigators were not completely blinded during the experiment.

Reporting for specific materials, systems and methods

We require information from authors about some types of materials, experimental systems and methods used in many studies. Here, indicate whether each material, system or method listed is relevant to your study. If you are not sure if a list item applies to your research, read the appropriate section before selecting a response.

Materials & experimental systems

n/a	Involved in the study	
☐	☒	Antibodies
☒	☐	Eukaryotic cell lines
☒	☐	Palaeontology and archaeology
☐	☒	Animals and other organisms
☐	☐	Human research participants
☒	☐	Clinical data
☒	☐	Dual use research of concern

Methods

n/a	Involved in the study	
☒	☐	ChIP-seq
☒	☐	Flow cytometry
☒	☐	MRI-based neuroimaging

Antibodies

Antibodies used	6C5 and 4B10
Validation	6C5 and 4B10 were generate by immunizing BAL/c mice with inactivated E30 F-particle and they can recognize, bind and neutralize E30 potently.

Animals and other organisms

Policy information about studies involving animals; ARRIVE guidelines recommended for reporting animal research

Laboratory animals	Three-week-old SPF BALB/c mice were used. The male and female mice were the same.
Wild animals	Not Applicable
Field-collected samples	Not Applicable
Ethics oversight	Jiangsu Provincial Center for Disease Control and Prevention

Note that full information on the approval of the study protocol must also be provided in the manuscript.

Human research participants

Policy information about studies involving human research participants

Population characteristics	Not Applicable

April 2020

Recruitment	Not Applicable
Ethics oversight	Not Applicable

Note that full information on the approval of the study protocol must also be provided in the manuscript.
